# Effectiveness of Herbal Mouthwash among Visually Impaired Residential School Students

**DOI:** 10.31729/jnma.3654

**Published:** 2018-08-31

**Authors:** Sirjana Dahal, Ashish Shrestha, Tarakant Bhagat

**Affiliations:** 1 Department of Community and Public Health Dentistry, Kathmandu Medical College Teaching Hospital, Duwakot, Bhaktapur, Nepal; 2 Department of Public Health Dentistry, B.P. Koirala Institute of Health Sciences, Dharan, Nepal

**Keywords:** *gingivitis*, *mouthwash*, *plaque control*, *randomized clinical trial*, *visually impaired*

## Abstract

**Introduction:**

Visually impaired patients are unable to visualize the plaque on tooth surfaces resulting in inadequate plaque removal and therefore the progression of dental caries and inflammatory disease of the periodontium. The objective of the study was to assess the effectiveness of herbal mouth wash in reduction of plaque and gingivitis among visually impaired children.

**Methods:**

Randomized controlled clinical trial was conducted with parallel groups study, comprising 6 to 20 year old visually impaired children, 20 in each group (herbal mouth wash or chlorhexidine mouthwash or placebo mouthwash). Plaque and gingival index were recorded at baseline and at the end of the study. Children were asked to use the mouth wash twice daily for two weeks. Analysis was done using Chi-square test for categorical data and Mann-Whitney U test/independent t-test and one way analysis of variance/Kruskal-wallis H test for quantitative data. The level of significance was set at P<0.05.

**Results:**

Participants showed fair oral hygiene (mean plaque scores of 1.14±0.53) and moderate gingivitis (mean gingival scores of 1.12+0.45) with no significant difference between three groups (P=0.47 and 0.84, respectively). Significant reduction of plaque and gingivitis was seen at follow-up with no significant difference between herbal and chlorhexidine mouthwash. However, significant difference was found between placebo and herbal/chlorhexidine mouthwash.

**Conclusions:**

Herbal mouthwash showed significant effect on reducing plaque formation and gingivitis in visually impaired students. The effectiveness of herbal mouthwash was analogous to the gold standard chlorhexidine.

## INTRODUCTION

Plaque control is a critical component of dental practice that permits long term success of periodontal and dental care.^1^ In addition to mechanical plaque control methods, efforts have been focused on chemotherapeutic agents for reducing or preventing plaque-induced oral diseases.^2^ Mouth rinses are agents that provide significant benefits to patients who cannot maintain adequate mechanical plaque control.^1^

Visually impaired patients are unable to visualize the plaque on tooth surfaces resulting in inadequate plaque removal, progression of dental caries and inflammatory disease of the periodontium.^3^ Chlorhexidine is considered as gold standard mouthwash but it has shown several side effects like staining and taste alteration, which limit its long term use.^4^ Therefore, different herbs are being widely explored to discover alternatives to synthetic antibacterial agents.^5^

This study was conducted to assess the effectiveness of herbal mouth wash and also to compare the effect of different mouthwashes in reduction of plaque and gingivitis among visually impaired children.

## METHODS

A randomized controlled clinical trial with parallel groups study was implemented in between December 12 to December 26, 2016 among visually impaired residential students of Dharan, Nepal. Ethical approval for the study was obtained from the Institutional Review Committee, BPKIHS, Dharan (Ref. No. 159/073/074-IRC). The study was registered as a clinical trial (www.ctri.nic. in) by the National Institute of medical Statistics (India Council of Medical Research); the Clinical Trial Registry India identifier no. CTRI/2017/03/008049 http://ctri.nic.in/Clinicaltrials/main1.php?EncHid=10853.39355.

A total of 82 visually impaired students of age 4 to 20 years were examined from Shree Purwanchal Gyanchakshu Vidhyalaya, Dharan, Nepal at baseline. Those who met the inclusion criteria were included in the study. Informed consent was obtained from the students under trial.

### i. Inclusion criteria

- Visually impaired students of age 6 to 20 years with minimum of 20 teeth present.
- Patient diagnosed with mild to moderate type of gingivitis.
- Patients who had not received any periodontal therapy except oral prophylaxis for the past six months.

### ii. Exclusion criteria

- Subjects taking antibiotics or any other drugs within last three months.
- Medically compromised subjects.
- Smokers.
- Patients who had periodontal pockets in excess of 4 mm/clinical attachment loss.
- Partial dentures or clinically unacceptable restorations or bridges.
- Patient with orthodontic appliances/undergoing orthodontic treatment.
- Patient with a known history of allergy to chemical or any herbal products.

Sample size was calculated by reference taken from a study carried out in Raichur, India^6^ among 100 participants with mean difference = 0.983 ± 0.355 (mean difference of before and after treatment with herbal mouth wash±standard deviation).


$$\begin{array}{l} \text{SE }=\text{ SD}/\sqrt{\text{n}}\\ \text{Or, }{\text{SE}}^{2}={\text{SD}}^{2}/n\\ \text{Or, n = }{\text{SD}}^{2}/{\text{SE}}^{2} \end{array}$$

Where,

SE= Standard error of mean
SD= standard deviation
n= sample size

Putting values in above formula,


$$\begin{array}{l} {\text{SE}}^{2}\;=\;{\left(0.355\right)}^{2}/100\\ =0.0013 \end{array}$$

Then,

n= (0.355)^2^/0.0013

Or, n= 96.942


$$\begin{array}{lll} \text{Corrected sample size} & = & \frac{\text{calculated sample size}}{1\;+\;\frac{\text{calculated sample size}}{\text{Estimated population}}}\\ & = & \frac{96\text{.942}}{1+\;\frac{96.942}{20}}\\ & & =\;\frac{96.942}{1+\;4.847}\\ & & =\;16.58 \end{array}$$

Adding 20% attrition rate, final sample around 20 in each group.

A randomization master list was prepared based on computer generated random numbers (in Microsoft Office Excel 2007) and each child was assigned to a group (Group 1 or Group 2 or Group 3). An assistant, not participating in the field study prepared the mouthwash (1:1 dilution) and packed in three identical opaque bottles for each mouthwash which were coded as follows:

Group 1: Herbal mouth wash.
Group 2: Chlorhexidine mouth wash (as positive control).
Group 3: Placebo mouth wash (as negative control).

The participants, their caregivers, examiner and analyzer were blinded to the treatment allocation throughout the trial.

The baseline and follow-up study visit after 2 weeks involved a full-mouth oral examination of all the teeth using the Plaque and Gingival index.^7,8^ The examiner was trained and calibrated prior to initiation of the study and during the study. Intra-examiner reliability was assessed by re-examining 25 randomly selected participants. All the subjects were examined in the supine position under natural light. Oral examination was carried out by using sterilized instruments including mouth mirror, explorer and marked probe.

Participants enrolled in the trial received either herbal mouth wash (Hiora mouthwash regular, manufactured by the Himalaya Drug Company, Makali, Bangalore, India) or chlorhexidine mouth wash (Hexidine manufactured by ICPA health product Ltd.) or placebo used as mouthwash after 24 hours of scaling. Hiora is an alcohol free herbal mouthwash with key indegridents being *Salvadora persica* (Meswak), *Piper betle* (Nagavalli) and *Belleric myrobalan* (Bibhitaki). Hexidine mouthwash contains 0.2% chlorhexidine digluconate. Placebo mouthwash was distilled water with edible colour added making it a coloured water solution.

The three mouthwashes were placed in three opaque bottles and were coded as 1, 2 and 3 by a person who was not involved in the trial. The randomization codes were kept in a sealed envelope until the end of the follow-up period. The participants, their caregivers, examiner and supervisor were blinded to the treatment allocation throughout the trial.

Proper instructions were given to the students about the proper usage of mouth wash. Mouth wash was diluted with distilled water (1:1) and total of 20ml (10ml mouth wash+10ml distilled water) was used for rinsing. The investigator supervised the dosage of mouth-wash being used to ensure proper mouthwash use.

Statistical Package for Social Sciences, version 20 was used for statistical analysis. The level of significance was set at 0.05. Intra-examiner reproducibility for coding was measured by Cohen's kappa coefficient. Descriptive analysis was performed to summarize the clinical and socio-demographic characteristics of each group at baseline in order to assess how comparable the groups were at beginning of the study. Independent t-test was used for genderwise comparison of pre-intervention plaque and gingival scores. Differences in mean plaque and gingival scores between herbal, chlorhexidine and placebo groups were evaluated using ANOVA and tukey post hoc test when compared before the intervention and Kruskal-wallis U test followed by Mann-Whitney U test when compared after intervention depending upon the distribution of data. Wilcoxon sign rank test was used for pairwise comparison of pre and post intervention plaque and gingival scores.

## RESULTS

**Figure 1.**
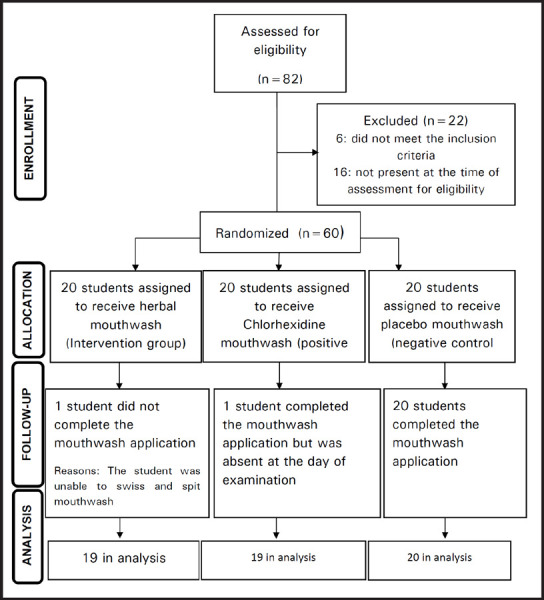
Flow chart of the study.

Out of 60 visually impaired students selected for the study 58 students completed the study (96.67% retention rate). Figure 1 presents the CONSORT flow diagram tracking the participation of students for the entire study. Mean age of the study participants was 12.7±4.07 years consisting of 34 (56.7%) males and 24 (43.3%) females. Intra-examiner reliability was assessed by overall kappa value which was 0.77 (substantial agreement) for plaque index and 0.81 (good agreement) for gingival index.

At baseline, the participants showed fair oral hygiene with mean plaque scores of 1.14±0.53 and moderate gingivitis with mean gingival scores of 1.12±0.45. Gender-wise comparison of plaque control and gingivitis at baseline depicted significantly higher plaque scores in males than females (P = 0.013). Baseline comparison of mean plaque scores in between herbal, chlorhexidine and placebo mouthwash was done using one way analysis of variance (ANOVA) which revealed no significant difference between three groups at baseline (Table 1). Table 2 displays the mean plaque and gingival scores in between three groups at baseline and after 14 days of intervention.

**Table 1:** Comparison of mean plaque and gingival scores of test and control groups at baseline.

Variables	Variance	Sum of squares	df	Mean square	F	P^†^
Plaque control at baseline	Between groups	0.43	2	0.21	0.74	0.47
	Within groups	16.52	57	0.29		
Gingivitis at baseline	Between groups	0.73	2	0.03	0.16	0.84
	Within groups	12.29	57	0.21		

* Significant

† *One way ANOVA*

Pairwise comparison of mean plaque and gingival scores between baseline and follow-up in herbal, chlorhexidine and placebo mouthwash showed significant reduction in both plaque and gingival scores (P<0.01) after 14 days of mouthwash use following scaling and root planing (Table 2). Results indicated that there was excellent plaque control (median=0) and mild gingivitis (median=0.46) after 2 weeks intervention.

**Table 2:** Pairwise comparison of mean plaque and gingival scores between baseline and follow-up in herbal, chlorhexidine and placebo mouthwash.

	Number of participants	Mean rank	Median	P^†^
Plaque control				
Baseline	58	31.39	1.14	<0.01^*^
Follow-up		15.71	0.00	
Gingivitis				
Baseline	58	30.60	1.10	<0.01^*^
Follow up		9.33	0.46	

* *Significant*

* *†Wilcoxon signs rank test*

**Table 3:** Comparison of plaque and gingival scores of test and control groups after two weeks of intervention.

Variables	Group	No. of participants	Mean rank	P^†^
Plaque scores	1	19	26.18	0.002^*^
	2	19	23.13	
	3	20	38.70	
Gingival scores	1	19	24.05	0.006^*^
	2	19	24.66	
	3	20	39.28	

* *Significant*

† *Kruskal-Wallis H test; 1=herbal group; 2= chlorhexidine group; 3=placebo group*

Significant difference in between the groups both with regard to plaque control and gingivitis (P = 0.002 and P=0.006, respectively) was seen at the time of follow-up (Table 3). Further investigation revealed significant difference in between plaque and gingival scores in between herbal mouthwash group and placebo group (P = 0.007 and 0.005, respectively). Similarly similar difference was seen between chlorhexidine and placebo mouthwash group (P=0.001 and 0.005, respectively). However no significant difference was seen between herbal and chlorhexidine mouthwash group (P=0.491 and P = 0.907, respectively) (Table 4).

**Table 4:** Comparison of plaque and gingival scores of test and placebo mouthwash groups after 2 weeks of intervention.

Variables	Group	No. of participants	Mean rank	P^†^
	Herbal group	19	15.68	0.007^*^
	Placebo group	20	24.10	
	CHX group	19	14.63	0.001^*^
Plaque control	Placebo group	20	25.10	
	Herbal group	19	20.50	0.491
	CHX group	19	18.50	
	Herbal group	19	14.76	0.005^*^
	Placebo group	20	24.98	
	CHX group	19	14.95	0.007^*^
Gingivitis	Placebo group	20	24.80	
	Herbal group	19	19.29	0.907
	CHX group	19	19.71	

* *Significant*

† *Mann-Whitney U test*

## DISCUSSION

Development of bacterial biofilm in the marginal gingiva and periodontal pockets has been attributed to be the main etiologic factor in the development and progression of periodontal disease.^9–11^ Mechanical plaque control methods are widely used traditionally in all parts of the world but evidence suggests that these methods are inadequate.^12^ Moreover, in visually impaired people the situation is even worse as they are at a higher risk of developing oral diseases especially periodontal disease because they have greater difficulty in achieving good oral hygiene.^13,14^ There is growing consensus that antiplaque agents can be used as adjuncts to mechanical cleaning.^12^

The present research is an attempt to investigate the effect of herbal mouth wash on reduction of plaque and gingivitis as an adjunct to scaling and root planning in visually impaired residential students. In this study, a commercially available herbal mouthwash containing different plant extracts: *Salvadora persica, Piper betle, Belleric myrobalan* was used as an experimental mouthwash; chlorhexidine mouthwash as positive control and coloured water solution as negative control.

The age group selected in this study was 6 to 20 years. Nevertheless, there was no significant difference in the mean age of participants in between three groups. The students selected for the study were visually impaired and from the same residential school wherein same food was served for all children. More than 20% of attrition was taken into account considering their disability limitation in cognitive learning. However, there was overall attrition of the sample by 3.33%. Among the two students who dropped out, one from herbal group could not continue the study as she was unable to swish and spit the mouthwash. The other student from placebo group completed the daily mouthwash rinse for two weeks but was absent at the day of final examination.

The participants had fair plaque control and moderate gingivitis at baseline examination. There was no significant difference in between three groups in plaque and gingival scores at pre-rinsing stage. Males showed significantly higher plaque scores than females indicating that females were more concerned about their oral hygiene maintenance than males. However, in comparison between groups no significant difference was found with regard to gender. Hence, the population selected for each of the three groups was homogenous. An experimental period of two weeks was chosen for mouthwash rinse after scaling and root planning concerning the fact that prolonged use of chlorhexidine mouthwash for more than 2 weeks causes tooth staining.^15^ However, the side effects of herbal mouthwash is not known.^6^

The results of the present trial demonstrated that there was significant reduction in plaque and gingival scores from baseline to two weeks of daily supervised mouthwash rinse used as an adjunct to normal oral hygiene procedures. In spite of several differences in methodological procedures, these findings are in accordance with those of various studies done in India in which herbal mouthwashes were used in order to assess their effectiveness in plaque control and gingivitis.^2,6,11,16,17^

The results of group I (herbal mouthwash group) indicate that the plaque and gingival scores at follow-up were lower (0.37±0.49 and 0.43±0.38, respectively) than that at the baseline (1.12±0.49, 0.37±0.49 respectively) suggesting improvement from fair to good plaque control and moderate to mild gingivitis after regular usage of mouthwash. This reduction in the plaque and gingival scores could be attributed to the antimicrobial, antiplaque and anti-inflammatory properties of *Salvadora persica^13,19^*' and also antimicrobial and anti-inflammatory properties of *Piper betle* (Nagavalli).^20^

Students in group II used chlorhexidine mouthwash (0.2%) in 1:1 dilution. The participants in this group also showed decline in plaque and gingival scores from the baseline (1.25±0.61 and 1.17±0.46 to 0.26±0.45 and 0.45±0.38, respectively) which is in accordance to other similar studies.^16,21^ These findings of chlorhexidine group can be ascribed to the mechanism of action of chlorhexidine in which the cationic molecule binds to the negatively-charged cell walls of the microbes, destabilizing their osmotic balance causing concentration-dependent growth inhibition and cell death. Also, secondary interactions causing inhibition of proteolytic and glycosidic enzymes may be significant.^22^

Students in group III used coloured water solution as placebo mouthwash. The students in this group also showed reduction in plaque and gingival scores at follow-up. However, the plaque scores were not significantly reduced from the baseline (P = 0.057). The reduced scores may be attributed to the scaling which was performed 24 hours before the mouthwash rinse was started.

Some adverse effects were reported by the participants using chlorhexidine mouthwash. Taste was regarded as bad by many of the participants of chlorhexidine group. On 8^th^ day of trial, one female participant reported ulceration on the tip of tongue and the other on anterior 1/3^rd^ of the tongue. However, the ulceration subsided within 24 hours and the mouthwash rinse was continued. Further complications were not seen. Burning sensation of tongue was reported by a male student at the time of rinsing. Bitter taste, burning sensation and ulceration are the side effects of chlorhexidine mouthwash.^23–25^

When an intergroup comparison was made between three groups at the follow-up period, the three groups showed statistically significant difference in both plaque and gingival scores (P=0.002 and P=0.006, respectively). On further analysis, herbal and chlorhexidine groups were significantly different from the placebo mouthwash group suggesting that the mechanical action of rinsing alone is not sufficient for the control of plaque and reduction of gingivitis. On the other hand herbal and chlorhexidine mouthwash showed no significant difference in plaque control and gingivitis (P = 0.491 and P = 0.907, respectively). From this result, it could be suggested that herbal mouthwash containing *Salvadora Persica* (Meswak), *Piper Betle* (Nagavalli) and *Belleric myrobalan* (Bibhitaki) was comparable to chlorhexidine in maintaining proper plaque control and healthy status of the gingiva.

There are some limitations of the study. The probable confounding effect of other independent factors have not been addressed. Supervision of daily mouthwash rinse was done but brushing twice daily with the proper technique was instructed to the participants and was assumed that they followed the instructions. Supervision from the teachers was considered acceptable. Additionally, participants who are enrolled in oral hygiene studies usually tend to improve their oral hygiene practice, irrespective of the product they receive. Even though the participants of the present study were not aware of the type of mouth wash they were provided with, another crucial factor is the novelty effect, which play a role in motivation of oral hygiene practice by the use of a new substance.

## CONCLUSIONS

The study results showed that herbal mouthwash potentially possesses a significant effect on reducing plaque formation and gingivitis when used as an adjunct to scaling and root planning in visually impaired students. The effectiveness of herbal mouthwash was analogous to the gold standard chlorhexidine. This mouthwash is palatable and well accepted by the differently abled children. Furthermore, longitudinal studies under controlled conditions for longer duration are required to establish the antiplaque and antibacterial effects of herbal mouthwash in people with special needs.
